# Evaluation of the Nova StatSensor^®^ Xpress^TM^ Creatinine Point-Of-Care Handheld Analyzer

**DOI:** 10.1371/journal.pone.0122433

**Published:** 2015-04-17

**Authors:** Cara Simone Kosack, Wim de Kieviet, Kubra Bayrak, Anastacija Milovic, Anne Laure Page

**Affiliations:** 1 Diagnostic Network, Médecins sans Frontières, Amsterdam, Netherlands; 2 Department of Clinical Chemistry, Sint Lucas Andreas Ziekenhuis, Amsterdam, Netherlands; 3 Epicentre, Paris, France; Virgen Macarena University Hospital, School of Medicine, University of Seville, SPAIN

## Abstract

Creatinine is a parameter that is required to monitor renal function and is important to follow in patients under treatment with potentially toxic renal drugs, such as the anti-HIV drug Tenofovir. A point of care instrument to measure creatinine would be useful for patients monitoring in resource-limited settings, where more instruments that are sophisticated are not available. The StatSensor Xpress Creatinine (Nova Biomedical Cooperation, Waltham, MA, USA) point of care analyzer was evaluated for its diagnostic performance in indicating drug therapy change. Creatinine was measured in parallel using the Nova StatSensor Xpress Creatinine analyzer and the Vitros 5,1FS (Ortho Clinical Diagnostics, Inc, Rochester, USA), which served as reference standard. The precision (i.e., repeatability and reproducibility) and accuracy of the StatSensor Xpress Creatinine analyzer were calculated using a panel of specimens with normal, low pathological and high pathological values. Two different Nova StatSensor Xpress Creatinine analyzers were used for the assessment of accuracy using repeated measurements. The coefficient of variation of the StatSensor Xpress Creatinine analyzers ranged from 2.3 to 5.9% for repeatability and from 4.2 to 9.0% for between-run reproducibility. The concordance correlation agreement was good except for high values (>600 µmol/L). The Bland-Altman analysis in high pathological specimens suggests that the Nova StatSensor Xpress Creatinine test tends to underestimate high creatinine values (i.e., >600 µmol/L). The Nova StatSensor Xpress Creatinine analyzers showed acceptable to good results in terms of repeatability, inter-device reproducibility and between-run reproducibility over time using quality control reagents. The analyzer was found sufficiently accurate for detecting pathological values in patients (age >10 year) and can be used with a moderate risk of misclassification.

## Introduction

Creatinine is a parameter that is required to monitor renal function. This parameter is important to follow in patients under treatment with potentially toxic renal drugs, such as the anti-HIV drug Tenofovir [1–4]. HIV programs are often located in decentralized settings where no laboratory is available to carry out blood analysis. The use of potentially toxic renal drugs in these settings leads to the demand for point-of-care analyzers for appropriate patient management in resource-limited settings.

The StatSensor Xpress Creatinine analyzer (Nova Biomedical Cooperation, Waltham, MA, USA) is a handheld Creatinine analyzer for the quantitative measurement of creatinine in capillary, venous, and arterial whole blood.

The instrument can operate at a temperature of 15 to 40 °C and at a relative humidity level of 10 to 90%, which makes it potentially suitable for typical low-resourced settings in Africa and other parts of the world.

To date no evaluations of the point-of-care analyzer StatSensor Xpress Creatinine analyzers has been published. All publications in peer reviewed journals have been performed on the StatSensor Creatinine analyzers and these showed varying results [5–8]. Two evaluations on StatSensor Creatinine analyzers showed an overall negative bias when compared to laboratory based measurements [5,6]. One study showed a linear measurement range up to 863 μmol/L [6]. A third study showed an increase of the coefficients of variation (CVs) up to 6-fold when the StatSensor Creatinine analyzers was used on whole blood compared to plasma methods [7], which could not be confirmed by another study [6]. In order to help with the decision whether or not to use this point of care analyzer to monitor creatinine in low-resourced settings, we evaluated the precision, accuracy and ability of the test to indicate appropriate therapeutic change.

## Methods

### Study design

This study was a prospective laboratory-based case-control evaluation of the diagnostic test precision and accuracy of Nova StatSensor Xpress Creatinine analyzer when compared to the reference method (Vitros 5,1FS, Ortho Clinical Diagnostics, Inc. Rochester, USA). The study was carried out at the Clinical Laboratory of the Sint Lucas Andreas Ziekenhuis (SLAZ), Amsterdam, Netherlands.

### Samples

Blood samples were drawn by venous puncture in lithium heparin containing tubes (BD Vacutainer, BD-Plymouth PL6 7BP, UK) from patients and co-workers of the SLAZ after taking informed consent from each participant.

Samples with normal creatinine concentrations were obtained from outpatients and co-workers, whereas samples with high creatinine concentrations were obtained from patients of the dialysis department.

All sampled tubes were kept close to prevent leakage of oxygen. All measurements on the Nova StatSensor Xpress Creatinine analyzers were carried out within 15 minutes after collection (except for the stability testing) using a syringe to transport the sample from the tube to the reagent strip.

### Test platform and procedure

Three Nova StatSensor Xpress Creatinine point-of-care analyzers (catalogue number 48635; Nova Biomedical Cooperation, Waltham, MA, USA) were used in this evaluation. The creatinine method of the Nova StatSensor Xpress Creatinine analyzer is an enzymatic method with amperometrical detection of the generated H_2_O_2_. Calibration was factory encoded in the reagent strip. The required sample volume of the StatSensor Xpress Creatinine is 1.2 μL and the measurements range is 27 to 1,056 μmol/L (i.e. 0.30 to 12.0 mg/dL). The assay was performed by two laboratory technicians following manufacturer’s recommendations. The laboratory technicians were blinded to the results of the reference methods.

### Reference method

Creatinine measurements by the Vitros 5,1FS (Ortho Clinical Diagnostics, Inc, Rochester, USA) analyzer using the Vitros Crea Slide method (Creatinine amidohydrolase / sarcosine oxidase / peroxidase method) served as reference standard. The dynamic range of this method is 4 to 1,200 μmol/L. This method is traceable to the National Institute of Standards and Technology (NIST) SRM 914 creatinine and calibrated with standard reference material. Testing was carried out according to the manufacturer’s instructions. All measurements were carried out in duplicate and the mean was calculated.

### Sample size for analytical performance

#### Sample stability

In order to assess stability of the Xpress measurement on Li-heparin anti-coagulated blood, one blood sample with high Creatinine level (i.e. >177μmol/L or >2 mg/dL) was measured 5 times on all three Nova StatSensor Xpress analyzers and in duplicate on the reference method within 30 minutes after venous puncture.

At time points 60, 90, 120, 150, 180, 210 and 240 minutes after venous puncture, the measurements were performed on all three analyzers in duplicates.

#### Precision

Precision was assessed by evaluating the repeatability (i.e. repeated measurements in the same conditions) and reproducibility (i.e. repeated measurements on consecutive days).

Repeatability was assessed by measuring the creatinine value of 9 specimens (3 specimens each with normal (<115 μmol/L), low (115 to 270μmol/L) and high (>270μmol/L) pathological values) 8 times on each of the 3 analyzers.

The reproducibility was assessed using the Nova StatSensor quality control reagents repeated every day for 11 consecutive days.

#### Accuracy

The accuracy of the 3 Nova StatSensor Xpress Creatinine analyzers was measured using the 5 Nova linearity solutions with known creatinine concentrations ranging from 88–900 μmol/L.

In addition, the accuracy Nova StatSensor Xpress Creatinine analyzers was evaluated by measuring 60 specimens including 20 with normal (<115 μmol/L), 20 with low pathological (115 to 270μmol/L) and 20 with high pathological (>270μmol/L) values on two different analyzers and in duplicate with the reference method.

### Ease of use

Two experienced laboratory technicians reported on the ease of use of the Nova StatSensor Xpress Creatinine analyzer.

### Data collection and statistical analysis

Data was collected using Excel (Microsoft Office, USA). Analysis was carried out using Excel (Microsoft Office, USA) and STATA version 12.1 (StataCorp, College Station, Texas, USA). Analyse-it Software Ltd (Leeds, UK) was used to perform the Bland-Altman agreement statistics and to calculate the Bland-Altman difference plots.

The performance of the Nova StatSensor Xpress Creatinine analysers was compared with the CLIA criteria for acceptable performance [9–11] and with the more stringent Westgard desirable biological variation database specifications [12].

#### Stability

Stability was assessed by calculating the coefficient of variation (CV = standard deviation/mean) of repeated measurements over the 4h-period for each analyzer separately.

#### Precision

For each specimen and each analyzer, the mean, standard deviation and CV of the repeated testing were calculated. According to the ‘Westgard Desirable Biological Variation Database Specifications’, the precision was found acceptable if the mean measurements had a CV of less than <8.9% for all time points [12].

In addition, the coefficient of reproducibility of the measurements of the 60 patients specimens on two different analyzers were calculated as suggested by Bland and Altman by calculating the standard deviation of the differences of the repeated measurements [13,14].

#### Accuracy

When measuring accuracy using linearity solutions, the measurement results of each Nova StatSensor Xpress Creatinine analyzer were plotted on a graph against the known creatinine concentration of the linearity solutions. Concordance with the expected values was measured for each analyzer using the concordance correlation coefficient, which combines measures of both precision and accuracy to determine how far the observed data deviate from the line of perfect concordance [10]. A concordance correlation coefficient r>0.95 was considered good, whereas r>0.80 was considered acceptable.

When evaluating the accuracy using patient specimens, the mean results of the Nova StatSensor Xpress Creatinine analyzer evaluation were compared to the mean results of the reference standard using Bland-Altman analysis to calculate a) the bias (i.e. mean of the difference) with 95% CI and b) the limits of agreement [bias +/- (1.96*standard deviation)]. The bias and the limits of agreement were compared with the CLIA criteria for acceptable performance for creatinine.

In addition, the measurements of the Nova StatSensor Xpress Creatinine analyzers vs. reference method were plotted on a correlation graph and the concordance correlation coefficient was calculated. A correlation coefficient of r>0.95 (R^2^>0.90) was considered good, whereas r>0.80 (R^2^>0.64) was considered acceptable.

### Impact on creatinine clearance

In clinical decision making for initiation and monitoring of patients on anti-retroviral therapy (ARVs) in HIV patients, creatinine clearance is used rather than crude creatinine value. The formula for creatinine clearance by Cockcroft-Gault equation is used when for adults weighing 50–75 kg. The Cockcroft-Gault equation for creatinine clearance (Cr CL) in mL/min is 1.23 x weight x (140—age/creatinine in μmol/L) for males and 1.04 x weight x (140—age/creatinine in μmol/L) for females.

Critical decision-making points for dose adjustments of Tenofovir are Cr CL values at 50, 30 and 10 mL/min. Two patient examples were simulated to illustrate the influence of the creatinine range for normal and high pathological values.

### Ethical statement

The study was submitted to Sint Lucas Andres Hospital ethical review board and has been carried out after their approval. Each study participant gave written consent to carry out the study on their specimens. The study was carried out in accordance with the Declaration of Helsinki concerning medical research in humans.

## Results

### Stability of creatinine in heparin samples

The specimen used to study the stability showed a mean value of 299.6 μmol/L using the reference method. The creatinine values in the three analyzers ranged from 276.5 to 312.0 μmol/L and did not vary substantially over the 4-hour period with coefficients of variations of 3.43, 2.41, and 3.40 in the three analyzers, respectively. These results allowed pursuing the evaluation with specimens tested within 4 hours of specimen collection.

### Precision

When assessing repeatability, the CV of the StatSensor Xpress Creatinine analyzers ranged from 5.9% in the normal creatinine range to 2.3% in the high pathological range (Table 1).

**Table 1:** Mean measurements in μmol/L, standard deviation and coefficient of variation of 8 repeated tests per specimen and per Nova StatSensor Xpress Creatinine analyzer.

		StatSensor 1			StatSensor 2			StatSensor 3			
		mean	SD	CV %	mean	SD	CV %	mean	SD	CV %	mean CV %
**Normal range**				5.6			7.6			4.7	5.9
	Sample 1	62.6	3.96	6.3	64.1	4.97	7.8	60.1	3.04	5.0	6.4
	Sample 2	58.8	2.25	3.8	59.3	3.80	6.4	57.9	2.03	3.5	4.6
	Sample 3	55.0	3.62	6.6	56.4	4.87	8.6	57.1	3.14	5.5	6.9
**Low pathological range**				3.8			4.4			3.6	3.9
	Sample 1	271.1	6.40	2.4	275.3	7.32	2.7	270.8	8.50	3.1	2.7
	Sample 2	169.9	6.70	3.9	172.8	7.44	4.3	170.4	7.82	4.6	4.3
	Sample 3	302.5	15.61	5.2	296.8	18.61	6.3	289.9	8.60	3.0	4.8
**High pathological range**				2.5			2.1			2.3	2.3
	Sample 1	695.3	21.50	3.1	696.1	21.31	3.1	702.6	15.30	2.2	2.8
	Sample 2	637.6	12.42	2.0	640.3	7.53	1.2	633.6	13.53	2.1	1.8
	Sample 3	372.0	8.58	2.3	374.3	7.89	2.1	369.4	9.66	2.6	2.3
**Mean CV%per analyser**				**4.7**			**5.5**			**4.2**	**4.1**

When assessing between run reproducibility over an 11-day period using the quality control reagents the CV ranged from 4.2 to 9.0% (Table 2) and all measurements were within the ranges given by the manufacturer.

**Table 2:** Mean measurements, standard deviation and coefficient of variation of 11 daily testing of quality control reagent per reagent and per Nova StatSensor Xpress Creatinine analyzer.

	StatSensor 1			StatSensor 2			StatSensor 3		
	mean	SD	CV %	mean	SD	CV %	mean	SD	CV %
**Level 1**	97.4	5.64	5.8	96.7	4.84	5.0	92.2	5.93	6.4
**Level 2**	210.3	12.9	6.1	211.5	9.89	4.7	200.9	18.1	9.0
**Level 3**	597.6	26.7	4.5	611.6	25.7	4.2	583.0	37.7	6.5

Using patient specimens, the reproducibility coefficient for the 2 Nova StatSensor Xpress Creatinine analyzers was 14.3 μmol/L overall, 2.9 μmol/L in the normal range, 11.6 μmol/L in the low pathological range, 20.8 μmol/L in the high pathological range.

### Accuracy

Using linearity solutions from Nova in the range from 88 to 900 μmol/L (i.e. 1.0 to 10.2 mg/dL), each of the 3 Nova StatSensor Xpress Creatinine analyzers showed good linearity with concordance coefficient greater than 0.99 (Table 3).

**Table 3:** Results of the measurements of the 5 linearity reagents per Nova StatSensor Xpress Creatinine analyzer and corresponding correlation coefficient.

	StatSensor 1	StatSensor 2		StatSensor 3
**Creatinine concentration (**μ**mol/L)**				
**88**	88	99		99
**203**	229	214		179
**398**	378	415		399
**601**	588	606		623
**866**	895	908		804
**Concordance coeffficient**	0.997	0.997		0.993

Nova StatSensor Xpress Creatinine analyzer 1 and 2 were used for the assessment of accuracy using repeated measurements by two different Nova StatSensor Xpress Creatinine analyzers for the comparison with the reference standard.

**Fig 1:**
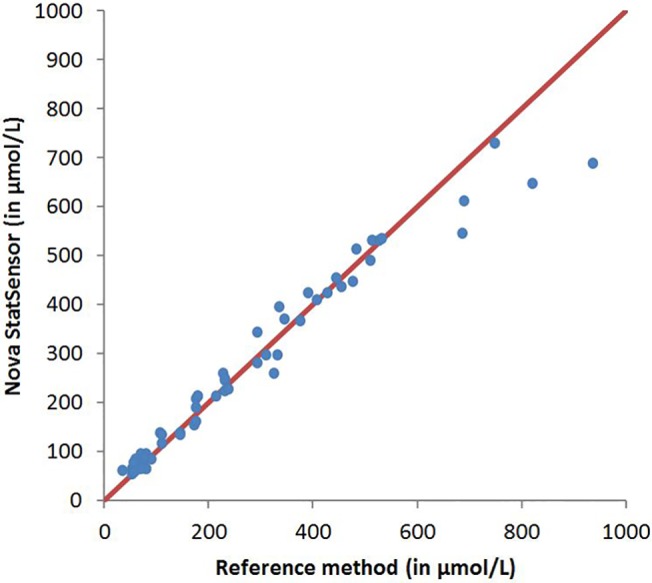
Scatter plot of Nova StatSensor Xpress Creatinine analyzer vs. Vitros 5,1FS (Ortho Clinical Diagnostics, Rochester, USA) measurements.

Using patient specimens, the graphic representation of the comparison of the means of repeated measurements on the two analyzers and the reference method with the y = x line shows that the agreement is good except for high values (>600 μmol/L with the reference method), where the Nova StatSensor Xpress Creatinine analyzers seem to reach a plateau (Fig 1).

Accordingly, the concordance correlation coefficient was 0.97 overall and went up to 0.99 if restricted to samples with a reference value <600 μmol/L (i.e. <6.8 mg/dL). The concordance correlation coefficients were 0.69, 0.90, and 0.83 for normal (<115 μmol/L), low pathological (115 to 270 μmol/L) and high pathological range (270 to 600 μmol/L), respectively.

**Fig 2:**
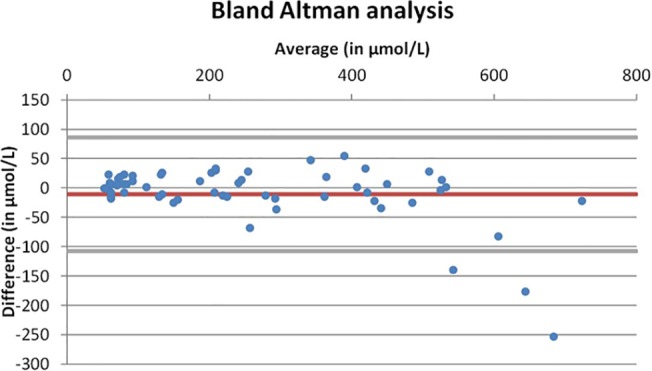
Bland Altman analysis of the accuracy of Nova StatSensor Xpress Creatinine analyzer vs. Vitros 5,1FS (Ortho Clinical Diagnostics, Rochester, USA) using duplicate measurements.

The Bland-Altman graphic representation of the overall results is shown in Fig 2 and the stratified results in the normal, low pathological, high pathological values are presented in Table 4.

**Table 4:** Summary of the Bland Altman analysis per type of specimen (normal range, low pathological, high pathological) and globally.

(in μmol/L)	Bias (95% CI)	Lower 95% LoA	Upper 95% LoA
**Normal range (N = 20)**	3 (-2.6;8.6)	-21.1	27
**Low pathological (N = 20)**	-4.3 (-16.6;8.0)	-56.7	48.1
**High pathological (N = 20)**	-30.9 (-67.4;5.7)	-187	125.3
**High pathological < 600** μ**mol/L (N = 15)**	4.5 (-9.9;19.0)	-46.7	56.7
**Total (N = 60)**	-10.7 (-23.5;2.0)	-109.6	88.2
**Total < 600** μ**mol/L (N = 55)**	0.8 (-5.2;6.7)	-43.2	44.8

As above, the trend shown in the Bland-Altman plot in high pathological specimens suggests that the Nova StatSensor Xpress Creatinine analyzers tend to underestimate high creatinine values (i.e. >600 μmol/L). When extremely high values of >600 μmol/L were excluded, the Bland Altman analysis among high pathological specimens was improved (Table 4).

### Misclassification

The Nova StatSensor Xpress Creatinine analyzers misclassified two specimens as pathological, which were considered ‘normal’ with the reference method. These two specimens were placed in the ‘low pathological group’ using the reference method and had reference values of 111 μmol/L and 113 μmol/L, very close to the threshold of 115 μmol/L used as the cut-off (Table 5).

**Table 5:** Cross-tabulation of Nova StatSensor Xpress Creatinine analyzers and reference method for classification in normal or pathological values (≥115 μmol/L).

	Nova StatSensor Xpress	
	Pathological	Normal
**Pathological values (≥115** μ**mol/L)**	38	0
**Normal values**	2	20

### Clinical decision making/creatinine clearance

We could not calculate creatinine clearance (Cr Cl) in our study population since we did not have access to age and sex of the patients. Instead, we simulated the Cr CL on two imaginary patients:

In the first example, we used a male patient of 30 years and a weight of 65 kg, with a true creatinine value of 80 μmol/L. In 95% of cases, the values measured by the index test (Nova StatSensor Xpress Creatinine analyzer) would be between 53 and 107 μmol/L. This would lead to a true Cr CL of 110 mL/min and measured values between 82 and 166 mL/min. If the true creatinine value was 200 μmol/L, measured creatinine would be between 143 and 257 μmol/L, leading to a true CR CL of 44 mL/min and measured Cr Cl between 34 and 61 mL/min. Table 6 illustrates the potential misclassification risk when using the Nova StatSensor Xpress Creatinine analyzer compared to the reference method modeled for this patient example (female, 30 years of age and 65 kg) using patients measurements from our study with a corresponding kappa of 0.85.

**Table 6:** Comparison of creatinine clearance results determined by the Nova StatSensor Xpress Creatinine analyzers or reference method modeled from the results obtained in the study applied to a hypothetical 65-kg, 30 year old male (N = 60).

		Nova StatSensor Xpress Creatinine			
		10–30 mL/min	30–50 mL/min	≥50 mL/min	Total
**Reference method**	<10 mL/min	1	0	0	1
	10–30 mL/min	21	2	0	23
	30–50 mL/min	0	10	2	12
	≥50 mL/min	0	0	24	24
	Total	22	12	26	60

In the second example, we used a female patient of 20 years of age and a weight of 50 kg, with a true creatinine value of 80 μmol/L. In 95% of cases, the values measured by the index test would be between 53 and 107 μmol/L. This would lead to a true CR CL of 78 mL/min and measured values between 58 to 118 mL/min. If the true creatinine value was 200 μmol/L, measured creatinine would be between 143 and 257 μmol/L. Table 7 illustrates the potential misclassification risk when using the Nova StatSensor Xpress Creatinine analyzer compared to the reference method using the results obtained modeled for this patient example (male, 20 years of age and 60 kg) with a corresponding kappa of 0.87.

**Table 7:** Comparison of creatinine clearance results determined by Nova StatSensor Xpress Creatinine analyzers or reference method modeled from the results obtained in the study applied to a hypothetical 50-kg, 20 year old female (N = 60).

		Nova StatSensor Xpress Creatinine				
		<10 mL/min	10–30 mL/min	30–50 mL/min	≥50 mL/min	Total
**Reference method**	<10 mL/min	3	2	0	0	5
	10–30 mL/min	0	25	0	0	25
	30–50 mL/min	0	2	6	0	8
	≥50 mL/min	0	0	2	20	22
	Total	3	29	8	20	60

### Ease of use

The Nova StatSensor Xpress Creatinine analyzer was perceived as practical and simple to perform. The package insert was found to be informative and simple to understand. No difficulties were encountered when using the analyzer.

## Discussion

### Stability

Creatinine was stable in whole blood samples collected in lithium-heparin blood collection tubes for 4 hours and gave similar results with the Nova StatSensor Xpress Creatinine analyzers during this period of time, which was an important result for our evaluation since the repetition of measurements implied that specimens were tested at different time points.

### Repeatability, reproducibility (inter-device and between-run)

All three Nova StatSensor Xpress Creatinine analyzers showed acceptable to good results in terms of repeatability (i.e. in same test conditions), inter-device reproducibility and between-run reproducibility over time using quality control reagents.

### Linearity

Linearity and concordance with reagents provided by the manufacturer and reported for the Nova StatSensor analyzer by Schnabl et al [6] were very good up to 866 μmol/L and 863 μmol/L respectively. In contrast the comparison of creatinine values with the reference method using patient specimens showed good linearity up to 600 μmol/L but a trend of Nova StatSensor Xpress Creatinine analyzers to underestimate high values (> 600 μmol/L), which could be due to a plateau phenomenon caused by the limitation of the enzyme capacity at high substrate concentrations. The plateau phenomenon is not observed using Nova solutions in the linearity check. This can be explained by the matrix differences between the Nova linearity solutions and the fresh human blood samples or by the possible existence of enzyme inhibitors in samples taken from dialysis patients. Despite this underestimation, the values obtained for these specimens using the Nova StatSensor Xpress Creatinine analyzers were >500 μmol/L and would thus all have been considered correctly as high pathological values.

### Comparison with the reference method and misclassification

According to the CLIA criteria for acceptable performance, the 95% limits of agreement of patient specimen measurement on the Nova StatSensor Xpress Creatinine compared to reference standard can be considered acceptable for values in the normal range, but not in the low pathological or high pathological values (Table 4). Overall, when considering only specimens <600 μmol/L, the 95% limits of agreement are slightly above the CLIA acceptable error of 40 μmol/L (i.e. 0.45 mg/dL) applicable for a medical decision threshold of 270 μmol/L, considered high pathological here [9].

For patients with normal values, limits of agreement between the measurements by Nova StatSensor Xpress Creatinine analyzers and the reference method were +/- 27 μmol/L, which is conform with the CLIA acceptable error of 27 μmol/L for normal values [9]. This means that 95% of patient samples with a true creatinine value of e.g. 80 μmol/L (normal value) will show values between 53 and 107 μmol/L using the Nova StatSensor Xpress Creatinine analyzer.

For patients with pathological values, the limits of agreement were +/- 57 μmol/L, which is not conform with CLIA acceptable errors of 40 μmol/L for a decision threshold of 270 μmol/L (i.e. 15% of 270 μmol/L) [9]. This would mean that 95% of patient samples with a true creatinine value of e.g. 200 μmol/L (low pathological value) will show values between 143 and 257 μmol/L using the Nova StatSensor Xpress Creatinine analyzer. However, this has limited consequences for clinical decision making as illustrated by the calculated creatinine clearances.

It is important to mention that the relatively large limit of agreement of +/-27 μmol/L in the normal range will give unacceptable results for children under 10 years old due to the lower reference value of <65 μmol/L. Therefore the analyzer should not be used in children <10 years of age.

Despite the large limits of agreement, the number of misclassified patients was limited, with the relative number of misclassified patients being acceptable, as shown by the fact that the only two patients were misclassified with values very close to the cut-off limit 115 μmol/L.

### Clinical decision making via creatinine clearance

The influence on the creatinine clearance and risk of misclassification via the creatinine clearance is mostly seen in patients with medium to high pathological values as the Nova StatSensor Xpress Creatinine analyzer tends to underestimate high values.

## Conclusion

In conclusion, the Nova StatSensor Xpress Creatinine analyzer was found reliable, but not equivalent to the reference method in terms of accuracy. The analyzer was found sufficiently accurate for detecting pathological values in patients (age >10 year) based on the limited number of misclassified patients in this cohort. In children <10 years of age, however, considering the lower reference values, the Nova StatSensor Xpress Creatinine analyser is not recommended.

In clinical practice, creatinine clearance is more important than the actual creatinine value specially when used to guide proper dosing of drugs that require adjustment with renal impairment. We specifically modeled our results to the renal dosing recommendations for the drug Tenofovir.

Our modeled results for 2 patients indicated a good agreement (i.e. kappa >0.80), and suggested that the Nova StatSensor Xpress Creatinine analyzer can be used for this classification with a moderate risk of misclassification. As for other biochemical measurements, the risk of misclassification around a fixed threshold should be taken into account and clinical decision should be made based on the overall clinical and biological context.
